# Echo-Bright Pericardium in Recurrent Idiopathic Pericarditis

**DOI:** 10.7759/cureus.85643

**Published:** 2025-06-09

**Authors:** Soomal Rafique, Amit Bhandari, Pragna Srinivas, Arisha Rafique, Avinash Murthy

**Affiliations:** 1 Internal Medicine, Southern Illinois University School of Medicine, Springfield, USA; 2 Internal Medicine, St. John's Hospital, Springfield, USA; 3 Internal Medicine, Rajarajeswari Medical College and Hospital, Bangalore, IND; 4 Internal Medicine, Liaquat University of Medical and Health Sciences, Hyderabad, PAK; 5 Cardiology, Prairie Heart Institute, Springfield, USA

**Keywords:** acute pericarditis, echo bright pericardium, idiopathic pericarditis, transthoracic echocardiogram, tte

## Abstract

Various radiological findings are observed in acute pericarditis, including pericardial thickening, pericardial effusion, and elevated filling pressures on transthoracic echocardiogram (TTE); thickened pericardium on cardiac CT; edematous pericardium depicted in T2 black-blood/short tau inversion recovery (STIR) images; and enhancement with late gadolinium enhancement (LGE) on cardiac MRI. However, the presence of a bright pericardium on TTE has seldom been reported. We report a case of acute pericarditis, diagnosed in a young female patient based only on typical chest pain and elevated inflammatory markers. Initially negligible, a bright pericardium was prominent on the recurrent episode after one year of the initial presentation.

## Introduction

Acute pericarditis is an inflammatory process involving the pericardium [1]. It accounts for 0.1% of hospital admissions and 5% of presentations with non-ischemic chest pain [2]. Ninety percent of the time, it's idiopathic or viral in etiology [3]. Acute pericarditis is traditionally diagnosed by the clinical presentation, pericardial friction rub, electrocardiogram (EKG) findings, and the presence of pericardial effusion on a 2D echocardiogram [3]. Additional imaging findings include an edematous, bright pericardium on T2 black-blood/short tau inversion recovery (STIR) MRI and enhancement on late gadolinium enhancement (LGE) sequences of cardiac MRI [4]. An echocardiogram has an important role in the diagnosis of acute pericarditis and usually shows pericardial effusion. Other findings include pericardial brightness, thickening, and abnormal septal motion [1]. We present a case of idiopathic acute pericarditis in which a transthoracic echocardiogram (TTE) demonstrated no evidence of pericardial effusion but revealed a bright pericardium, highlighting the significance of a previously described cardiac MRI finding - yet a rare echocardiographic observation - and underscoring the importance of transthoracic echocardiography in the definitive diagnosis of this pathology.

## Case presentation

A 20-year-old female with a history of anxiety and depression presented with severe, sharp substernal chest pain that began two days ago. The pain worsened when lying on her back and improved when leaning forward. She also reported chills, exertional dyspnea, and nausea, with the pain persisting but improving over the past two days. Her review of systems was otherwise negative, with no recent travel, sick contacts, oral contraceptive use, or recreational drug use. She was taking spironolactone for acne, and fluoxetine, rizatriptan, nortriptyline, and bupropion for depression and migraine. No recent changes in medications were observed.

Other than tachycardia, the examination was negative. On presentation, her blood pressure was 114/79 mmHg, heart rate 124 beats per minute, temperature 98.5°F, respiratory rate 15 breaths per minute, and O_2_ saturation 99% on room air. An EKG was initially done, which revealed sinus tachycardia and incomplete right bundle branch block, but no ST wave changes were observed.

On initial investigations, she was found to have elevated C-reactive protein and D-dimer levels; pulmonary embolism was ruled out with chest CT. Chest X-ray, troponin, and other workups were within normal limits, as shown in Table 1.

**Table 1:** Pertinent work-up CRP: C-reactive protein; ESR: erythrocyte sedimentation rate; TSH: thyroid-stimulating hormone; ANA: antinuclear antibody; dsDNA: double-stranded DNA antibody; CT: computed tomography; LVEF: left ventricular ejection fraction; RVEF: right ventricular ejection fraction

	During initial presentation	On recurrent episode (one year later)	Normal value
Hemoglobin (g/dl)	13.5	14	12-15
White cell count (×10^3^)	7.2	10.8	4-10
Platelets (×100^3^/ul)	216	280	150-400
Potassium (mmol/l)	3.4	3.8	3.5-5.3
Magnesium (mg/dl)	1.7	-	1.6-2.6
Creatinine (mg/dl)	0.84	1	0.5-1.02
Calcium (mg/dl)	7.9	-	8.5-10.1
Albumin (g/dl)	3.1	-	3.4-5
Total bilirubin (mg/dl)	0.6	-	0.2-1
High-sensitivity troponin (ng/l)	<3	3	0-53
Lipase (U/L)	105	-	0-160
CRP (mg/dl)	34	2.16	<0.8
ESR (mm/hr)	4	5	0-20
TSH (mlU/l)	0.896	-	0.35-3.7
ANA ratio	0.2	-	<0.7
dsDNA (IU/ml)	0.7	-	<10
Rheumatoid factor	<10	-	<15
Chest X-ray	Normal	Normal	-
CT chest	No acute cardiopulmonary abnormality noted.	-	
Cardiac MRI findings	Normal left ventricle cavity size and wall thickness. No regional wall motion abnormalities. Calculated LVEF 55%. Normal right ventricle cavity size. Calculated RVEF 55%. No evidence of significant valvular abnormalities, delayed enhancement, or pericardial effusion.		

With classic pleurisy symptoms, suspicion of pericarditis, and the negative work-up for secondary causes, a diagnosis of idiopathic pericarditis was made. The patient’s initial echocardiogram and cardiac MRI, however, were normal. She was started on colchicine 0.6 mg twice daily, which was switched to naproxen 250 mg twice daily due to intolerance. Discharged on day 3 with a Holter monitor, her follow-up showed a resolution of symptoms and a normal echocardiogram.

**Figure 1:**
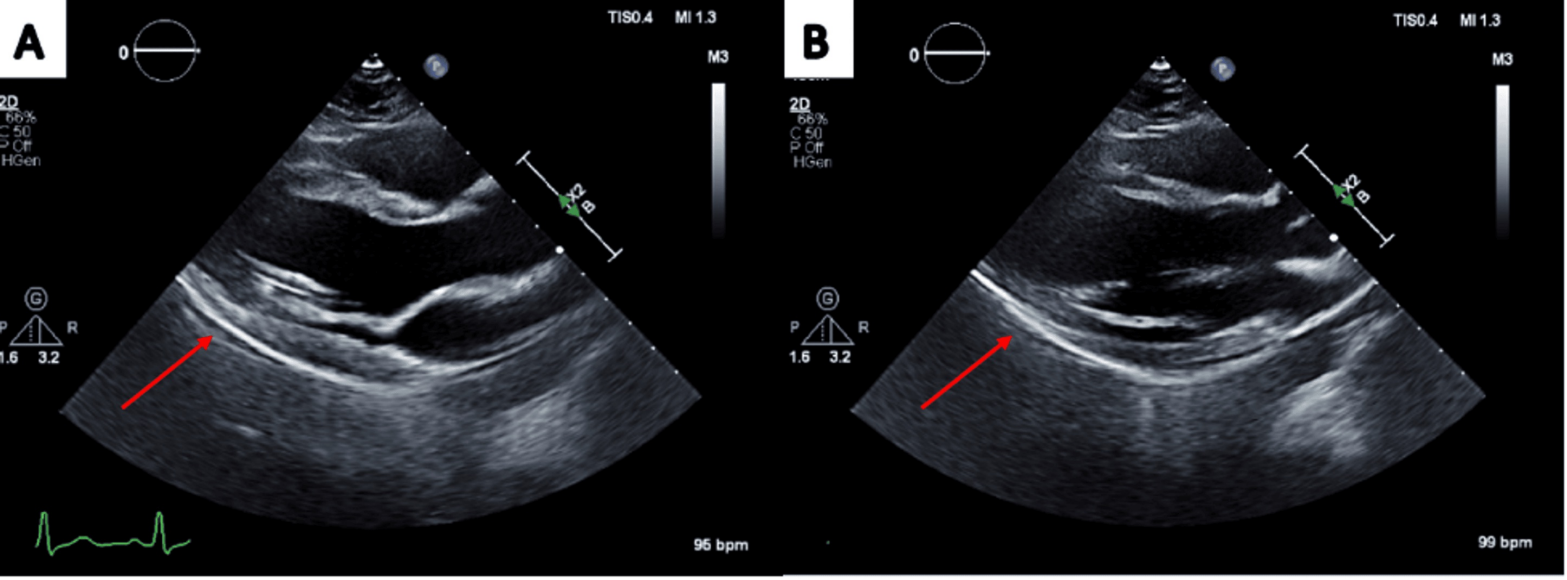
Transthoracic echocardiogram showing bright pericardium on parasternal long axis (PLAX) view (A and B) (red arrows)

One year later, she presented with similar chest pain, shortness of breath, and dizziness after upper respiratory symptoms. She was compliant with metoprolol tartrate 12.5 mg twice daily, which was started for sinus tachycardia. The patient started using naproxen 250 mg twice daily as needed, with no response. In the emergency room, she had elevated C-reactive protein. Managed with intravenous (IV) morphine and ketorolac, she was discharged with outpatient cardiology follow-up. In the clinic, she was started on a colchicine and nonsteroidal anti-inflammatory drug (NSAID) combination, which she tolerated well. A repeat echocardiogram revealed an echo-bright pericardium, as shown in Figures 1A-1B.

**Figure 2:**
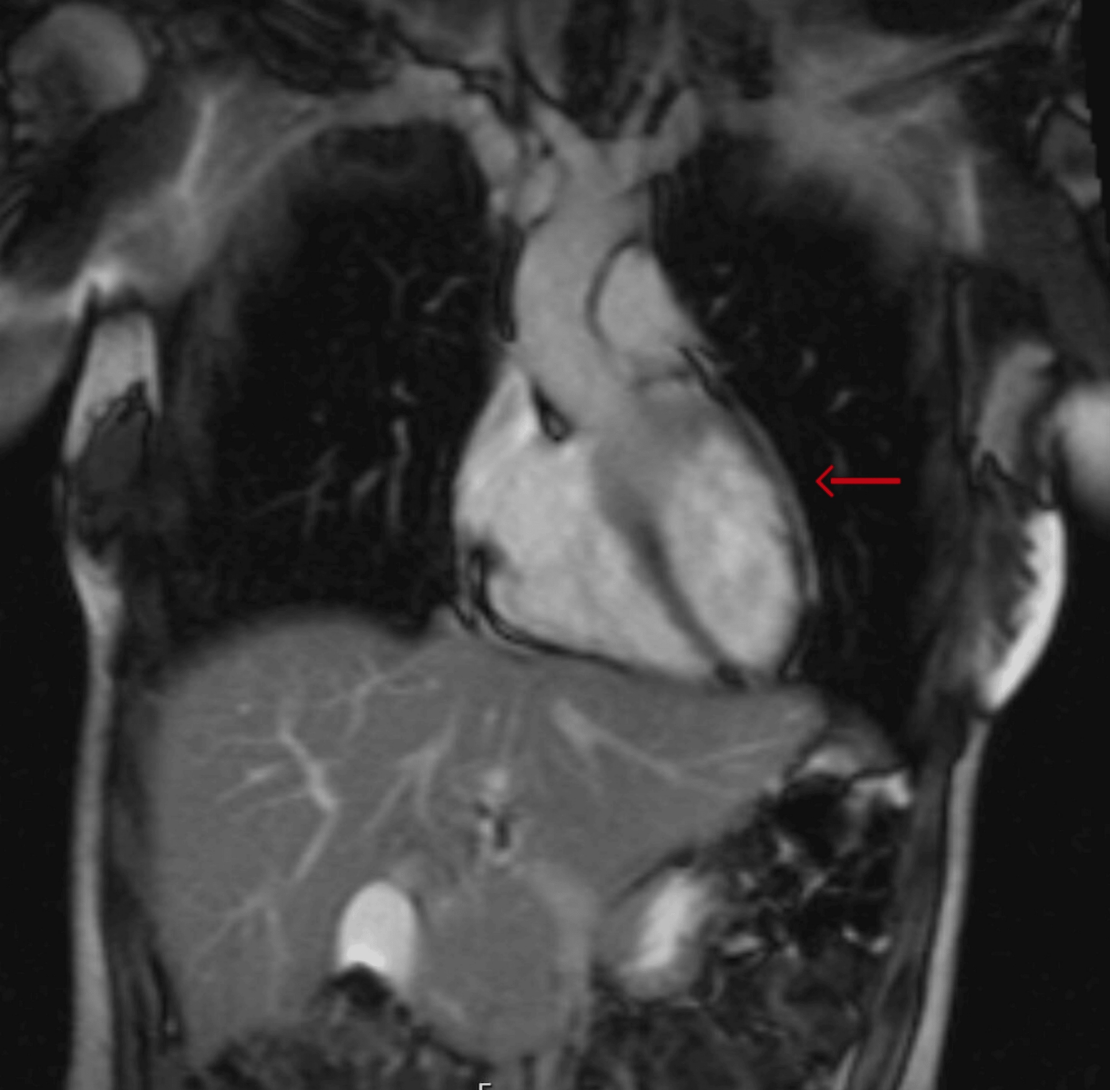
Cardiac MRI after two years, revealing normal pericardium (red arrow)

Subsequent follow-ups showed symptom control on colchicine alone and normal heart rate on metoprolol. However, serial cardiac MRI after two years of the initial episode did not reveal persistent pericardial inflammation (Figure 2).

Table 2 shows TTE and cardiac MRI findings over the course of illness.

**Table 2:** Transthoracic echocardiogram and cardiac MRI findings

	On initial presentation	Six months later	After one year-during the recurrent episode	Follow-up in clinic two years after the initial episode
Transthoracic echocardiogram findings	Left ventricular systolic function 64%. Wall motion normal in all segments. Normal right ventricular function. No significant valvular disease. No evidence of pericardial effusion.	Left ventricular systolic function 55%. Wall motion normal in all segments. Normal right ventricular function. No significant valvular disease. No evidence of pericardial effusion.	Estimated left ventricular ejection fraction is 55-60%. Right ventricular systolic function is normal. Trace mitral regurgitation. Echo bright pericardium noted. Sinus tachycardia.	
Cardiac MRI findings	Normal-sized chambers and wall thickness. No delayed enhancement or pericardial effusion.			Normal-sized chambers and wall motion and thickness. No delayed enhancement or pericardial effusion (as shown in Figure 2).

## Discussion

A normal pericardium is a 0.5 to 1 mm thick layer of connective tissue that is usually not visible on the echocardiography [4] but can be visualized as a highly echogenic line from its interface with lung tissue [5]. If the pericardial layer thickens more than 5 mm, it can be visualized on echocardiography. However, CT and cardiac magnetic resonance imaging (CMR) can often visualize normal pericardial thickness, where it appears as a thin, linear, intermediate-density layer surrounded by low-attenuation fat on CT [6] and a curvilinear line of low signal intensity between the myocardium and pericardial fat on both EKG-gated T1-weighted spin echo and gradient echo sequences on MRI [7]. Transesophageal echocardiography (TEE) can detect fluid pockets in the visceral layer recesses [4].

Recently associated with COVID-19 infection and vaccine [4], pericarditis is most commonly associated with viral infections or remains idiopathic, and its persistence beyond three months defines it as chronic [1]. The pericardium with a thickness greater than 2 mm is considered greater than normal pericardial thickening, which can be seen in acute or chronic pericarditis, with or without associated pericardial effusion [8].

Echocardiography in pericarditis usually shows pericardial effusion. Other findings include pericardial brightness, thickening, and abnormal septal motion reflecting evolving constriction. A bright or thickened pericardium is a less specific feature and can also be observed [1]. A CT scan can also reveal pericardial thickening by enhancing the thickened pericardial layers with the administration of the contrast and pericardial effusion, as well as differentiating fluid characteristics (exudate versus transudate) [1]. However, in those patients with negative echo and CT, cardiac MRI should be considered, which gives a detailed evaluation of the pericardium as well as the cardiac tissue [2]. On the CMR, pericardial thickening can be appreciated on the black blood and steady-state free precession (SSFP), and edema is detected as high-intensity on STIR imaging, whereas inflammation and fibrosis are evident by LGE [4]. The high-intensity signal on STIR imaging was found to have a moderate sensitivity of 63-68% in two studies and a high specificity of 100% in one study for pericarditis. LGE had moderate to high sensitivity of 65-100% across three studies and high specificity in two studies (99-100%) evaluating pericarditis [4].

Recurrent pericarditis affects about one-third of patients, often within the first 18 months [3]. Subsequent episodes may show less evident pericardial effusion and electrocardiographic changes compared to the initial episode [9]. Cardiac CT and MRI can reveal recurrent pericarditis through pericardial enhancement, inflammation in surrounding tissues, and blood in the pericardial space [1]. CMR can also be used to assess the degree of inflammation and to help guide treatment with anti-inflammatory agents, including interleukin-1 blockers. Chronic fibrotic pericarditis may present as pericardial thickening but without enhancement [4]. Progressive cases may present with pericardial irregularities on CT. In the chronic phase, the resolution of the STIR signal and decreased LGE severity correlate with recurrence and clinical remission [1].

The echocardiographic findings in acute pericarditis most commonly include pericardial effusion, and the echo-bright pericardium has been a rare and forgotten finding, which can also be seen in several other pathologies including, but not limited to, constrictive pericarditis and post-radiation changes. Since pleural effusion is expected to be seen on echocardiogram for the diagnosis of acute pericarditis, the pericardial brightness on echocardiogram has rarely been reported; therefore, it is not a novel but an under-reported finding. On the other hand, bright pericardium with STIR and LGE imaging on CMR, although moderately sensitive and specific, should also need caution with interpretation, and the quality, timing, and artifact effect should be taken into consideration. Prominent epicardial fat has been reported to mimic pericardial effusion [10], and pericardial effusion has, on the other hand, been found to be misleading in the diagnosis of left ventricular tear [11]. The pericardial calcifications from pericarditis, too, can reflect to give a false impression of intracardiac masses [12]. Increased gain can be thought to mimic pericardial brightness; however, it would result in generalized bright echo images, rather than focal pericardial enhancement, and further research is mandated to figure out other artifacts that can give a false impression of the echo-bright pericardium.

## Conclusions

Our case report describes a persistent echo-bright pericardium on TTE in a patient with recurrent idiopathic pericarditis, underscoring a rare yet potentially significant diagnostic clue with implications for both diagnosis and prognosis. While the ongoing presence of pericardial echo brightness may correlate with an increased risk of relapse, its detection in the absence of clinical symptoms necessitates careful clinical correlation and consideration of potential imaging artifacts. In cases with ambiguous clinical presentation or without evident pericardial effusion, the identification of a bright pericardium should prompt further imaging evaluation. Additionally, further studies are warranted to investigate the role of persistent pericardial echo brightness in predicting recurrence.
